# Supplementary figures and images for: Gut-Kidney Impairment Process of Adenine Combined with Folium sennae-Induced Diarrhea: Association with Interactions between Lactobacillus intestinalis, Bacteroides acidifaciens and Acetic Acid, Inflammation, and Kidney Function
Source: Cells. 2022 Oct 17;11(20):3261. doi: 10.3390/cells11203261 (PMC9599973; doi:10.3390/cells11203261)

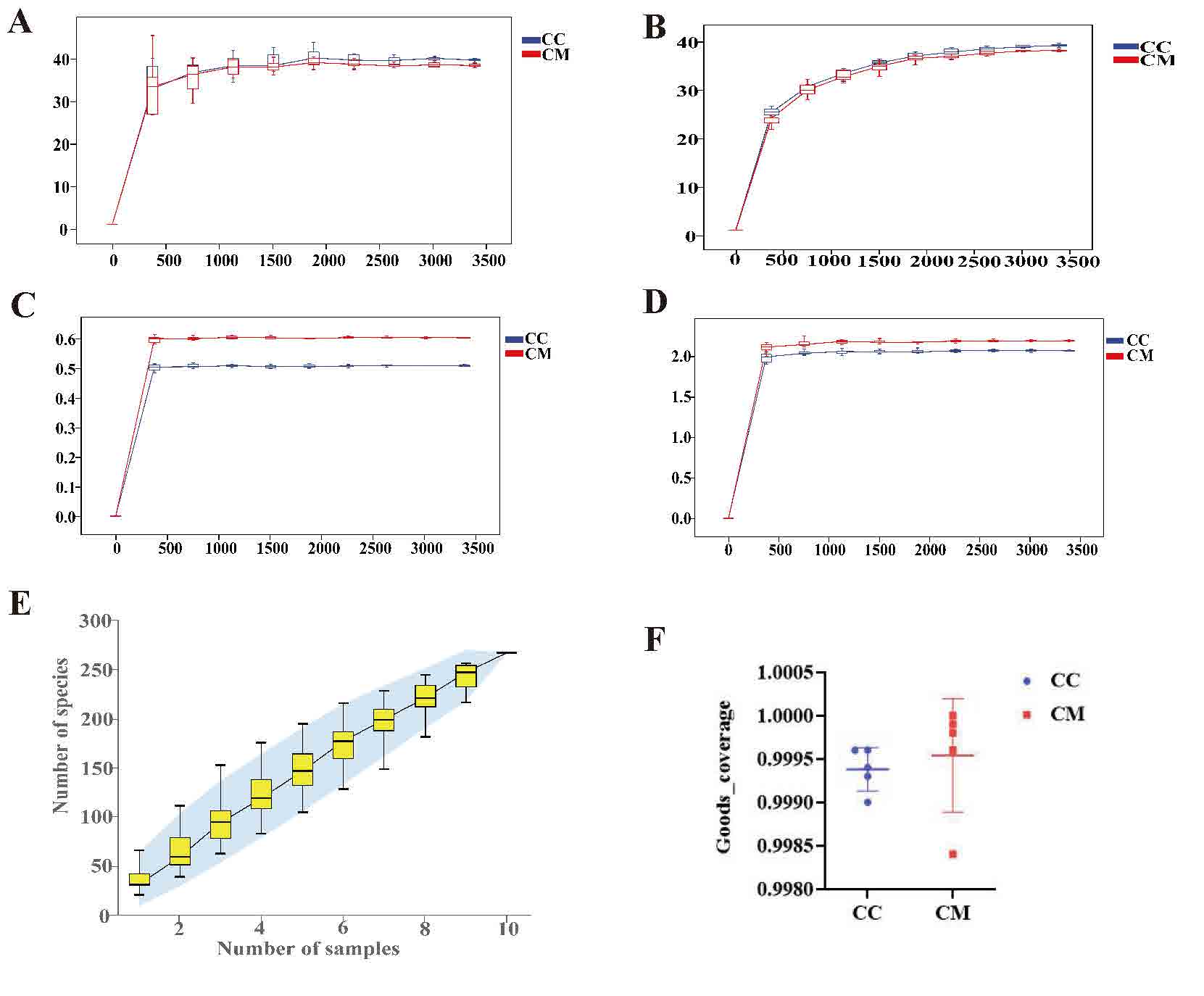

Supplement: Supplementary file 1 [file cells-11-03261-s001.zip › cells-1924242-Supplementary Figure S1.jpg]
